# Development and Modeling of a Novel Magnetorheological Elastomer Isolator in Hybrid Mode with a Compression–Shear Hybrid Fractional-Derivative Parametric Model

**DOI:** 10.3390/s25206376

**Published:** 2025-10-15

**Authors:** Yun Tian, Zhongwei Hu, Yingqing Guo, Lihua Zhu, Jun Dai, Yuxuan Tao, Xin Wang

**Affiliations:** 1 School of Civil Engineering, Xi’an University of Architecture and Technology, Xi’an 710055, China; 2 Institute of Dynamics and Smart Disaster Prevention, Northeastern University, Shenyang 110819, China; 3 College of Mechanical and Electronic Engineering, Nanjing Forestry University, Nanjing 210037, China; 4 China-Pakistan Belt and Road Joint Laboratory on Smart Disaster Prevention of Major Infrastructures, Southeast University, Nanjing 211189, China

**Keywords:** magnetorheological elastomer (MRE), hybrid mode MRE isolator, dynamic mechanical properties, compression-shear hybrid fractional-derivative parametric (CSHF) model, hybrid magnetic field generation system

## Abstract

Magnetorheological elastomers (MREs) are composed of soft silicone rubber, carbonyl iron particles (CIPs), and various additives. This study designs and fabricates a novel hybrid-mode MRE isolator that can operate in both compression and shear modes simultaneously. Experimental and modeling investigations are conducted to examine the dynamic mechanical properties of the hybrid-mode MRE isolator under varying excitation frequencies, displacement amplitudes, and magnetic field strengths. The equivalent stiffness, energy dissipation, and equivalent damping of the MRE isolator are determined. Experimental results reveal that the hybrid-mode MRE isolator exhibits a pronounced MR effect by utilizing a hybrid magnetic field generation system, with all three parameters significantly increasing as the magnetic field strength increases. However, as the excitation frequency increases, the equivalent stiffness and energy dissipation increase, while the equivalent damping decreases. Based on the experimental findings, a compression–shear hybrid fractional-derivative parametric (CSHF) model is proposed to describe the impact of different operating conditions on the dynamic viscoelastic properties of the MRE isolator. A comparative analysis of the experimental results and model predictions indicates that the proposed mechanical model can accurately describe the dynamic mechanical characteristics of the hybrid-mode MRE isolator.

## 1. Introduction

Magnetorheological elastomers (MREs) represent a class of advanced intelligent materials composed of a polymer matrix embedded with micron-scale ferromagnetic particles [1,2,3,4,5]. During the fabrication process, these particles, under the influence of an external magnetic field, align along the magnetic field lines to form chain-like or columnar structures, which, upon curing, result in anisotropic MREs [6,7]. Due to their rapidly tunable mechanical properties in the presence of magnetic fields—offering variable stiffness and damping—along with additional advantages such as particle stability (resistance to sedimentation), ease of storage, and no requirement for hermetic sealing, MREs have garnered increasing attention in active and semi-active vibration control technologies across diverse fields, including smart devices, aerospace, automotive, and civil engineering [8,9,10,11,12]. In these fields, it is common for components to withstand a static vertical preload while simultaneously mitigating horizontal (or vertical) vibrations, including seismic base isolation bearings, powertrain mounts, and human–machine interaction systems. In the realm of civil engineering, MRE-based dampers and isolators have sparked significant interest in recent years [13,14,15,16,17]. Scholars have conducted extensive research on various aspects of MRE isolators and other MRE applications, including design, experimental studies, and mechanical modeling [18,19,20].

For instance, Hu et al. [21] investigated the influence of MRE microstructural characteristics, as well as the macroscopic geometric parameters, on the magneto-mechanical response of MRE isolators. Then, a multi-scale computational method was developed by the same authors to describe the magneto-mechanical coupling effect of MRE isolators. Yu et al. [22] experimentally studied a phenomenological model to describe the hysteretic response of MRE isolation devices. Dynamic tests using a shake table demonstrated that the modified LuGre friction model outperformed other models. Moreover, Akhavan et al. [23], considering the effects of Casimir force and fringe fields, were the first to investigate the instability of an MRE-sandwiched cantilever MEMS actuator. Based on Euler–Bernoulli beam theory, the governing equations and associated boundary conditions were derived using the variational principle. Bao et al. [24] developed an MRE-based isolator and designed a semi-active fuzzy controller. Both numerical and experimental results showed that the controller effectively characterized the nonlinear behavior of the isolator and significantly reduced the relative displacement and absolute acceleration of scaled-down structures. The beneficial roles of semi-active fuzzy controllers in MRE-based isolators were also supported by other studies [25,26,27]. In addition, Aguib et al. [28] explored the nonlinear static behavior of MRE-sandwiched beams under transverse shear buckling. Further numerical and experimental studies were also conducted on the dynamic behavior of sandwich plates under the influence of magnetic fields [29]. In the application of MRE for vibration isolation, the primary operational modes are pure shear and pure compression. The shear mode offers the advantage of a broader tunable range for both stiffness and damping across specific frequency ranges, and its mechanical behavior is more readily modeled. Conversely, the compression mode provides a comparatively narrower adjustable range for stiffness and damping.

In addition, Lin et al. [30] proposed a novel MRE-based isolator, which exhibits adjustable stiffness, damping, and active control capabilities. Their study demonstrated that the transmission characteristics could be effectively modulated by the applied current and were strongly correlated with the amplitude of the acceleration excitation. Zhao et al. [31], leveraging the complementary effects of various materials, introduced a new approach to improve the vibration characteristics of sandwich panels, which could also be utilized in MRE-based isolators. At the same time, they proposed a unified modeling framework for functionally graded graphene-reinforced MRE (FGGR-MRE) sandwich panels. A nonlinear dynamic model of the sandwich panels was also established [32]. Furthermore, Bao et al. [33] introduced a new adaptive parameter identification method and further explored techniques for determining the nonlinear vibration structure parameters of MRE-based isolators. Using this theoretical approach, they demonstrated the asymptotic stability of response tracking and the robustness against dynamic excitation. Chen et al. [34] examined the buckling behavior of MRE composite laminates. The experimental results indicated that the critical buckling strain could be adjusted by an external magnetic field, and a computational model was developed to predict magneto-mechanical instability. In addition, Li et al. [35] found that increasing the magnetic field, the thickness ratio of the MRE layer to the overall structure, and temperature all contributed to the enhanced vibration-damping performance of MRE-functionalized composite plates. They also proposed a nonlinear analysis model that accounts for both internal magnetic and thermal fields. However, most studies have neglected to explore the combined effects of both operational modes. The challenge of harnessing the advantages of each mode while simultaneously optimizing the internal magnetic field strength within MRE isolators to maximize the magnetorheological (MR) effect remains a key area for future research [36,37,38].

This study presents the design and fabrication of a novel MRE isolator operating in a hybrid compression–shear mode and develops a corresponding system for dynamic mechanical performance testing (equivalent stiffness, energy dissipation, and equivalent damping). The relevance of this study to sensors manifests in three domains: magnetic field measurement, mechanical response acquisition, and model identification [39,40]. The shear mode enables a broad tunable range of stiffness and damping, while the compression mode provides a vertical load-bearing capacity and mitigates long-term creep. This parallelized configuration allows a trade-off between stiffness and damping without compromising preload stability. Building on the traditional compression–shear hybrid MRE isolator, a hybrid magnet system—integrating both electromagnets and permanent magnets—is introduced to achieve a superior zero-field effective stiffness without requiring an external current input, thereby enhancing the magnetorheological (MR) effect and expanding the adjustable stiffness range of the MRE isolator. The dynamic mechanical performance of the MRE isolator is evaluated using a universal testing machine under varying displacement amplitudes, frequencies, and magnetic field intensities. Furthermore, a compression–shear hybrid fractional-derivative parametric (CSHF) model is proposed to more accurately describe and predict the dynamic mechanical behavior of the MRE isolator. Another innovation point of this model is that it could explicitly capture the magnetic field and frequency dependencies of the MRE isolator with relatively few parameters. The experimental results are compared with the model’s calculated predictions to validate the accuracy of the proposed model.

## 2. Manufacturing of the Compression–Shear Hybrid-Mode MRE Isolator

### 2.1. Preparation of MREs

In this study, silicone rubber is utilized as the matrix material, while carbonyl iron particles (CIPs), with an average particle size of 3–5 μm, serve as the filler. The mass ratio of silicone rubber to CIPs is 6:4. The manufacturing process of the MREs can be summarized as follows: First, CIPs are mixed with liquid silicone rubber component A and thoroughly stirred for 2 min using an electric mixer. Subsequently, the same stirring process is employed to combine the mixture with silicone rubber component B. The resulting blend is then poured into a custom aluminum mold and degassed in a vacuum chamber for 5 min. The MREs are pre-structured and vulcanized simultaneously at 90 °C for approximately 60 min using a self-developed magneto-thermal coupling device, after which they are demolded for subsequent use.

### 2.2. Preparation of the Compression–Shear Hybrid-Mode MRE Isolator

**Figure 1:**
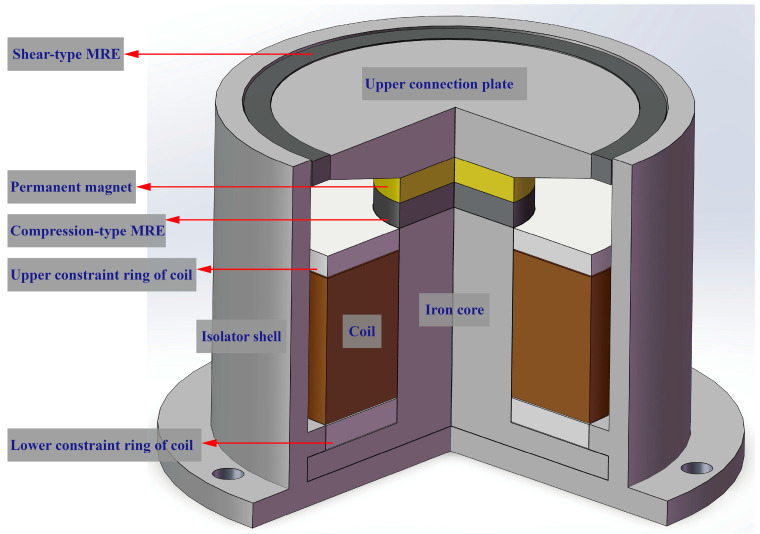
Schematic diagram of hybrid-mode MRE isolator structure.

**Figure 2:**
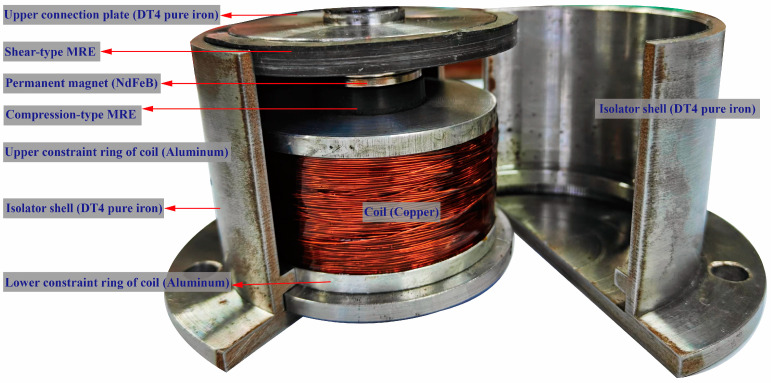
Hybrid-mode MRE isolator photo.

**Figure 3:**
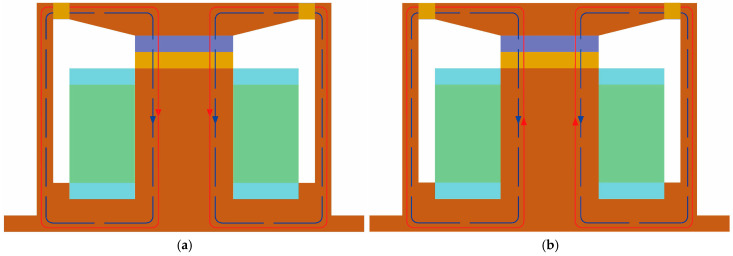
Hybrid magnetic system of hybrid-mode MRE isolator. (**a**) Under positive current; (**b**) under negative current.

The configuration of the novel compression–shear hybrid-mode MRE isolator developed in this study is depicted in Figure 1 and is primarily composed of nine components. As shown in Figure 2, the isolator shell, upper connection plate, and iron core are fabricated from DT4 pure iron, selected for its high magnetic permeability and stable properties. The upper and lower constraint rings of coil, constructed from non-magnetic aluminum, are affixed to the iron core and function mainly to partition the magnetic circuit and provide structural constraints at both coil terminals. The permanent magnet used is composed of neodymium–iron–boron, while the coil is made of 0.5 mm diameter enameled copper wire. The isolator shell, MRE, upper connection plate, permanent magnet, and iron core together constitute a closed magnetic circuit, as indicated by the red loop (generated by electromagnets) and the blue loop (generated by permanent magnets) in Figure 3. The silicone rubber-based MRE operates in two distinct modes: shear and compression. The dimensions of the shear-type MRE are 140 × 5 × 5 mm, while the compression-type MRE has a diameter of 30 mm and a thickness of 5 mm.

During the experiment, the upper connection plate oscillates vertically with the actuator head, inducing shear deformation in the shear-type MRE and compression or tensile deformation in the compression-type MRE. The primary advantages of the novel compression–shear hybrid-mode MRE isolator stem from the two aspects of “hybrid”: the hybrid of MRE deformation modes and the hybrid of permanent and electromagnetic magnets.

The isolator employs two MRE element types operating in parallel. The shear-type MRE exhibits a broader tunable range of stiffness and energy dissipation compared to the compression mode under varying magnetic field intensities and driving frequencies [41]. Conversely, the compression-type MRE deforms axially under compression and tension, offering enhanced load-bearing capacity and significantly mitigating the creep caused by prolonged static pressure within the system, thereby enhancing its potential for practical applications [42]. Furthermore, the inclusion of permanent magnets endows the isolator with increased stiffness and damping capabilities independent of the external power input, thereby reducing the energy consumption. The superposition and cancelation between the magnetic fields of permanent magnets and electromagnets extends the range of magnetic flux density and intensifies the magnetorheological (MR) effects of the MRE isolator. In practical deployments, the two modes are complementary: reverse-current cancelation is useful for calibrations, safety checks, and low-stiffness states, whereas co-directional superposition offers maximum stiffness and loss when isolation bandwidth or transmissibility limits demand it.

## 3. Experimental Analysis

### 3.1. Experimental Setup and Procedure

**Figure 4:**
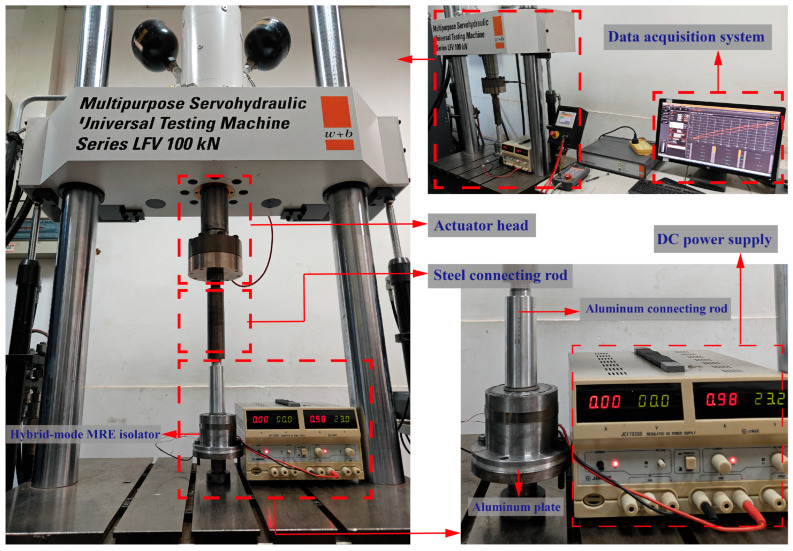
Experimental setup.

To characterize and assess the dynamic mechanical performance of the hybrid-mode magnetorheological elastomer (MRE) isolator, a series of experimental tests are conducted using a multi-purpose servo-hydraulic universal testing machine under varying load conditions [43]. The experimental setup is illustrated in Figure 4, with the testing machine capable of applying dynamic loads of up to ±100 kN. A DC power supply is used to provide an input current ranging from 0 to 2 A, with 0.5 A intervals, to regulate the magnetic flux density within the isolator. The magnetic flux density B of both the compression-type and shear-type MREs inside the isolator is measured using a Tesla meter. It is found that the superposition of two magnetic fields yields a flux density of 300–1200 mT in the compression MRE and 0–500 mT in the shear MRE. When a reverse current of 0.3 A is applied, the opposing magnetic fields in the compression MRE effectively cancel each other out, reducing the magnetic flux density to a negligible 16.7 mT. At this point, the mechanical performance of the MRE isolator could be considered its zero-field characteristic. This experiment utilizes a non-magnetic aluminum connecting rod and an aluminum plate to isolate the magnetic field within the MRE isolator from the upper steel connecting rod and the lower base attachment. This arrangement ensures that the magnetic field remains concentrated within the MRE isolator, preventing leakage due to the magnetic permeability of the connecting components, thereby preserving the MR effect of the MREs. In the following discussion, the magnetic flux density B is measured in the compression MRE to capture variations in its magnetic response.

In this part, a calibrated Tesla meter is used for in situ measurements of the magnetic flux density inside the device, which shows a great relevance to sensors, providing a traceable reference for field characterization and for parameterization under hybrid magnetic-circuit conditions [44,45].

This study employs a displacement control mode for dynamic testing, with the excitation and response signals recorded by the data acquisition system of the universal testing machine and subsequently transmitted to a computer for processing and analysis. To assess the dynamic viscoelastic properties of the hybrid-mode MRE isolator, cyclic sinusoidal excitation is applied at various loading frequencies and magnetic field strengths under constant displacement amplitudes. The experiment utilizes four typical displacement amplitudes (0.15, 0.25, 0.4, and 0.5 mm, corresponding to strains of 3%, 5%, 8%, and 10%), five loading frequencies (0.1, 0.3, 1.5, 3, and 5 Hz), and five magnetic flux densities (0, 300, 650, 950, and 1200 mT) for testing. As shown in Table 1, the experimental process comprises four stages, with ten cycles of data recorded at each frequency during each stage. For each frequency step, the first two cycles are discarded to eliminate transient effects. Then, the indicator parameters ($K_{e}$, $C_{e}$, and $E_{d}$) are calculated from the final eight cycles and then averaged to obtain a trial-level estimate. The data acquisition system operates at a sampling frequency of 1024 Hz, and tests are repeated twice under identical conditions. The final reported values represent the average of the two repeated runs. The real-time force–displacement data collected by the computer will be used to generate force–displacement hysteresis curves for future analysis. Also in this part, a synchronized force–displacement and current–voltage sensing chain delivers high-temporal-resolution excitation and response records, which enable a reliable estimation of the equivalent stiffness, energy dissipation, and damping, and support the identification and validation of the CSHF model parameters.

### 3.2. Results and Discussion

**Figure 5:**
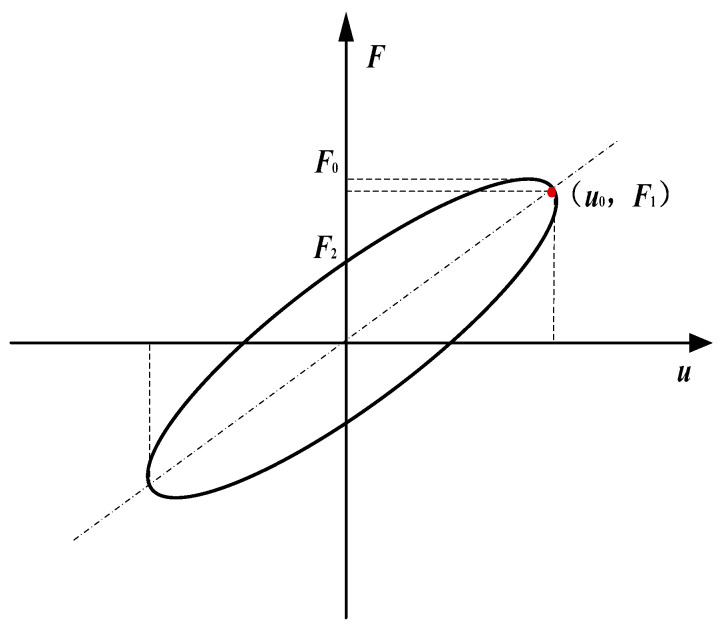
Characteristic force–displacement curve of MRE.

This study investigates the dynamic mechanical properties of the proposed hybrid-mode magnetorheological elastomer (MRE) isolator through a pre-designed experimental setup, focusing on equivalent stiffness ($K_{e}$), energy dissipation ($E_{d}$), and equivalent damping ($C_{e}$) characteristics. These indicator parameters could be calculated through the characteristic curve, as shown in Figure 5.

The equivalent stiffness ($K_{e}$) and equivalent damping ($C_{e}$) of MRE specimens could be directly calculated from the experimental data, and their specific expressions are as follows:

(1) $$K_{e}=\frac{F_{1}}{u_{0}}$$

(2) $$C_{e}=\frac{F_{2}}{\omega u_{0}}$$

where $u_{0}$ represents the maximum displacement of the MRE specimen, $F_{1}$ is the force corresponding to the maximum displacement $u_{0}$, $F_{2}$ denotes the force when the displacement is zero, and ω is the excitation frequency.

**Figure 6:**
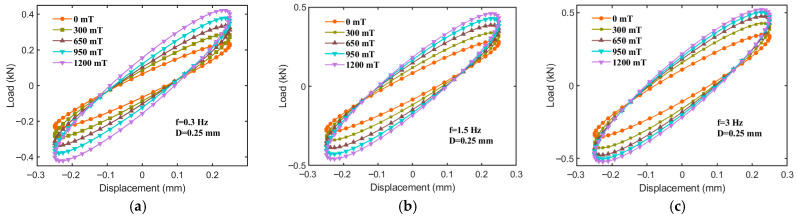
Force–displacement relationships for different magnetic fields at 0.25 mm. (**a**) f = 0.3 Hz, (**b**) f = 1.5 Hz, (**c**) f = 3 Hz.

As illustrated in Figure 6, under a fixed displacement amplitude of 0.25 mm and constant excitation frequencies of 0.3, 1.5, and 3 Hz, the force–displacement curves of the hybrid-mode MRE isolator exhibit relatively regular elliptical shapes as the applied magnetic field intensity varies from 0 to 1200 mT. Notably, the slope of the ellipse’s major axis increases significantly with a higher magnetic field intensity, indicating that the equivalent stiffness of the MRE isolator rises with an increase in magnetic field strength—demonstrating the MRE isolator’s excellent magnetorheological (MR) effect. This behavior is likely due to the enhanced magnetization of magnetic particles and the corresponding increase in interparticle attraction with rising magnetic field strength, resulting in increased equivalent stiffness. Another plausible explanation is that, under compressive deformation, the interparticle distance decreases and, due to magnetic attraction, may not fully revert to its original spacing upon recovery, thereby further strengthening interparticle forces and enhancing stiffness. Additionally, the area and fullness of the hysteresis loop (representing energy dissipation) increase markedly with magnetic field intensity, suggesting a pronounced effect of magnetic field strength on the MRE isolator’s damping characteristics, indicative of an MR effect of damping. In summary, these findings reveal that the proposed hybrid-mode MRE isolator exhibits distinctly adjustable stiffness and damping properties under an external magnetic field.

**Figure 7:**
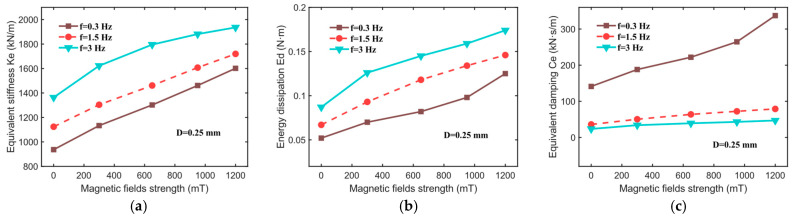
Mechanical properties of hybrid-mode MRE isolator at different magnetic fields. (**a**) Equivalent stiffness, $K_{e}$; (**b**) energy dissipation, $E_{d}$; (**c**) equivalent damping, $C_{e}$.

To further assess the influence of the magnetic field intensity on the equivalent stiffness ($K_{e}$), energy dissipation ($E_{d}$), and equivalent damping ($C_{e}$) of the hybrid-mode MRE isolator, these parameters are derived from the force–displacement curves shown in Figure 5 and plotted in Figure 6. As evident from Figure 7, the equivalent stiffness ($K_{e}$) of the MRE isolator increases significantly with escalating magnetic field strength. Specifically, at an excitation frequency of 0.3 Hz, the equivalent stiffness ($K_{e}$) rises from 936.64 kN/m (at 0 mT) to 1601.65 kN/m (at 1200 mT), reflecting a 71% enhancement due to the magnetorheological (MR) effect. Similarly, energy dissipation ($E_{d}$) increases from 0.052 N·m to 0.125 N·m, representing a 140% improvement, while the equivalent damping ($C_{e}$) rises from 141.19 kN·s/m to 337.19 kN·s/m. The substantial enhancements in these mechanical performance metrics under magnetic field stimulation further corroborate the strong MR effect of the fabricated hybrid-mode MRE isolator, underscoring its promising potential for future applications in vibration isolation and attenuation.

**Figure 8:**
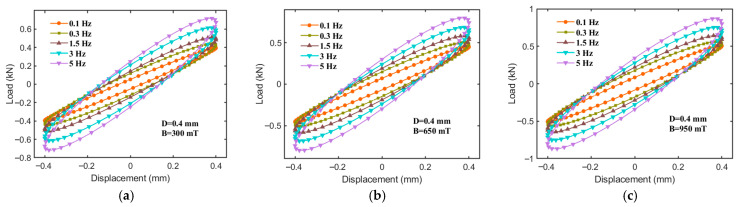
Force–displacement relationships for different frequency inputs at 0.4 mm. (**a**) B = 300 mT, (**b**) B = 650 mT, (**c**) B = 950 mT.

In addition, this study examines the impact of the excitation frequency on the equivalent stiffness ($K_{e}$), energy dissipation ($E_{d}$), and equivalent damping ($C_{e}$) of the hybrid-mode MRE isolator. As illustrated in Figure 8, under magnetic field intensities of 300, 650, and 950 mT, a fixed displacement amplitude of 0.45 mm, and within the frequency range of 0.1 to 5 Hz, the force–displacement curves of the MRE isolator also exhibit relatively regular elliptical shapes. From Figure 8, it is evident that the slope of the major axis of the hysteresis loop increases with a rising excitation frequency, and the area of the ellipse also expands progressively with frequency, indicating a typical frequency dependence in the dynamic mechanical behavior of the MRE isolator. This phenomenon may be attributed to the following factors: firstly, the viscoelastic nature of the MRE matrix itself exhibits frequency dependence, resulting in a superior stiffness at higher frequencies. Secondly, under high-frequency conditions, the duration of external force acting on the magnetic particle chains is reduced, making it more challenging to disrupt or rearrange the chain structures. Consequently, the interactions between magnetic particles remain stable, reducing the likelihood of relative slip and thereby enhancing the equivalent stiffness.

**Figure 9:**
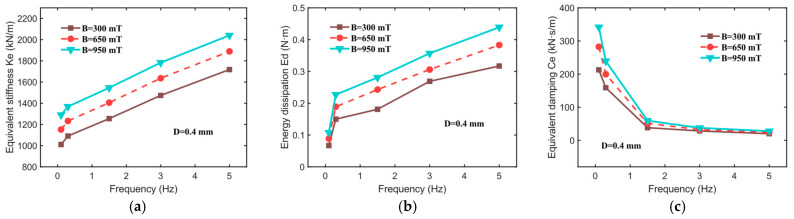
Mechanical properties of hybrid-mode MRE isolator at different frequencies. (**a**) Equivalent stiffness, $K_{e}$; (**b**) energy dissipation, $E_{d}$; (**c**) equivalent damping, $C_{e}$.

Figure 9 presents the trends in equivalent stiffness ($K_{e}$), energy dissipation ($E_{d}$), and equivalent damping ($C_{e}$) as functions of frequency. With an increasing excitation frequency, $K_{e}$ and $E_{d}$ show upward trends across all magnetic field intensities, but $C_{e}$ shows a downward trend. For instance, at a magnetic field intensity of 650 mT, the equivalent stiffness of the MRE isolator rises from 1152.03 kN/m at 0.1 Hz to 1889.33 kN/m at 5 Hz, an increase of 737.3 kN/m. Similarly, energy dissipation increases from 0.089 N·m to 0.383 N·m, while equivalent damping decreases from 282.559 kN·s/m to 24.256 kN·s/m. From a microstructural perspective, at low frequencies, field-induced particle chains and aggregates have sufficient time to undergo interfacial slip, rupture, and reformation relative to the matrix. This results in a greater rate-dependent viscous dissipation per cycle. As the frequency increases, the microstructural rearrangement cannot fully complete within each cycle; the interfacial sliding at both particle–matrix and particle–particle boundaries is reduced. Consequently, the phase lag between force and displacement is diminished ($C_{e}$ decreases), even though the absolute energy dissipated per cycle (i.e., the area of the hysteresis loop) may still increase with ω. Therefore, the effect of the excitation frequency should be taken into account when developing a mechanical model of the hybrid-mode MRE isolator.

**Figure 10:**
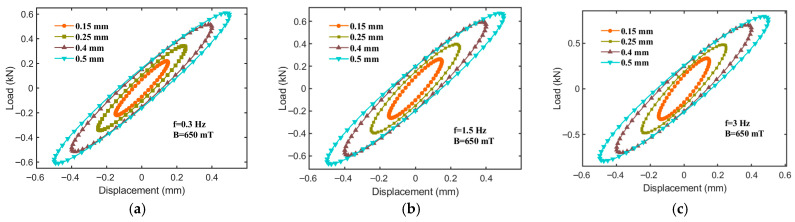
Force–displacement relationships for different displacement inputs at 650 mT. (**a**) f = 0.3 Hz, (**b**) f = 1.5 Hz, (**c**) f = 3 Hz.

To evaluate the effect of the displacement amplitude on the equivalent stiffness ($K_{e}$), energy dissipation ($E_{d}$), and equivalent damping ($C_{e}$), tests are conducted under a magnetic field intensity of 650 mT and constant excitation frequencies (0.3, 0.5, and 3 Hz) across various displacement amplitudes (0.15, 0.25, 0.4, and 0.5 mm). The resulting force–displacement relationships are shown in Figure 10. As illustrated, the slope of the ellipse’s major axis decreases with an increasing displacement amplitude, indicating that the equivalent stiffness ($K_{e}$) of the hybrid-mode MRE isolator declines as the displacement amplitude increases. This phenomenon may stem from two primary factors: first, at smaller displacement amplitudes, the magnetic particle chains within the MRE isolator experience minimal deformation, thereby maintaining stable mechanical properties. However, as the displacement amplitude increases, the particle chains undergo a greater deformation or even reorganization, leading to a reduction in the equivalent stiffness. Secondly, larger displacement amplitudes may induce the stretching or compression of molecular chains within the MRE matrix, potentially causing chain breakage or restructuring, which further reduces the isolator’s equivalent stiffness.

**Figure 11:**
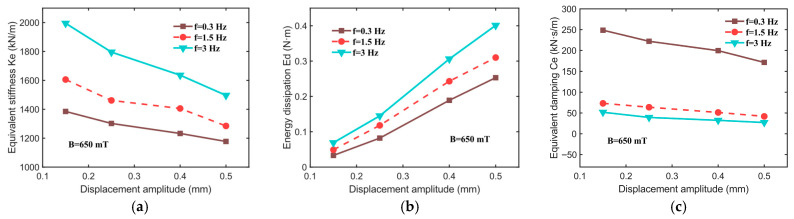
Mechanical properties of hybrid-mode MRE isolator at different displacement amplitudes. (**a**) Equivalent stiffness, $K_{e}$; (**b**) energy dissipation, $E_{d}$; (**c**) equivalent damping, $C_{e}$.

Figure 11 illustrates the declining trend in equivalent stiffness ($K_{e}$) and equivalent damping ($C_{e}$) as the displacement amplitude increases; however, energy dissipation ($E_{d}$) increases significantly as the displacement amplitude increases. For instance, at an excitation frequency of 1.5 Hz, $K_{e}$ decreases from 1605.66 kN/m (at 0.15 mm) to 1284.53 kN/m (at 0.5 mm), $E_{d}$ increases from 0.049 N·m to 0.31 N·m, and $C_{e}$ reduces from 73.17 kN·s/m to 41.87 kN·s/m.

**Figure 12:**
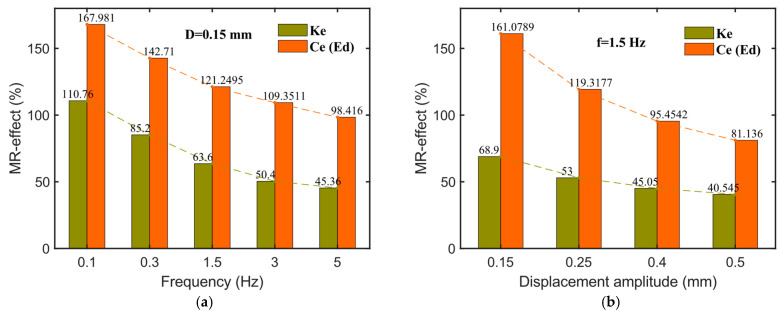
Relative MR effect of hybrid-mode MRE isolator at different loading conditions. (**a**) At different frequencies; (**b**) at different displacement amplitudes.

To further illustrate the influence of the excitation frequency and displacement amplitude on the relative magnetorheological (MR) effect of various mechanical performance indicators in the hybrid-mode MRE isolator, bar charts are generated, as shown in Figure 12, with the relative MR effect defined according to Equation (3). Figure 12a presents the impact of different excitation frequencies (0.1–5 Hz) on the MR effect at a fixed displacement amplitude of 0.15 mm. As shown in Figure 11a, the relative MR effect on energy dissipation ($E_{d}$) or equivalent damping ($C_{e}$) (relative MR effect is the same for these two parameters) is significantly more pronounced than its effect on equivalent stiffness ($K_{e}$). Moreover, with increasing frequency, the relative MR effect for all three indicators shows a downward trend. For example, the relative MR effect on $K_{e}$ decreases from 110.76% at 0.1 Hz to 45.36% at 5 Hz, while $E_{d}$ (or $C_{e}$) decreases from 167.981% to 98.461%. This trend may be attributed to the increased stability of magnetic particle chain structures within the MRE as excitation frequency rises, which enhances the zero-field modulus and consequently reduces the relative MR effect. Figure 12b depicts the effect of the displacement amplitude on the relative MR effect at a constant frequency of 1.5 Hz. As the displacement amplitude increases (0.15, 0.25, 0.4, and 0.5 mm), the relative MR effect for each performance metric gradually declines. For instance, the relative MR effect on $K_{e}$ decreases from 68.9% at 0.15 mm to 40.545% at 0.5 mm, while $E_{d}$ (or $C_{e}$) decreases from 161.079% to 81.136%.

(3) $$Relative\;MR\;effect=\frac{\left(M_{emax}-M_{e0}\right)}{M_{e0}}\times 100\%$$

where $M_{e0}$ denotes the initial equivalent stiffness $K_{e0}$ (or equivalent damping $C_{e0}$) when the magnetic field intensity is 0 mT, and $M_{emax}$ is the maximum equivalent stiffness $K_{emax}$ (or equivalent damping $C_{emax}$) at the highest magnetic field intensity (1200 mT in this article).

## 4. Mathematical Modeling

### 4.1. Compression–Shear Hybrid Fractional-Derivative Parametric Model

The experimental results indicate that, when the displacement amplitude remains within the linear viscoelastic (LVE) strain range, the force–displacement curve of the compression–shear hybrid-mode magnetorheological elastomer (MRE) isolator forms an approximately complete elliptical shape. This phenomenon can be interpreted as the superposition of the shear mechanical behavior and the tensile–compressive mechanical behavior of the MREs. Upon applying a magnetic field to the compression–shear hybrid-mode MRE isolator, the equivalent stiffness ($K_{e}$), energy dissipation ($E_{d}$), and equivalent damping ($C_{e}$) exhibit significant variations with changes in the magnetic field, demonstrating excellent magnetorheological (MR) properties. Moreover, the experimental data reveal that the mechanical performance of the MRE isolator is influenced not only by the external magnetic field but also by the loading frequency and displacement amplitude. To accurately describe and predict these behaviors, a compression–shear hybrid fractional-derivative parametric (CSHF) model, based on the parallel configuration of the tensile–compressive and shear components, is proposed.

**Figure 13:**
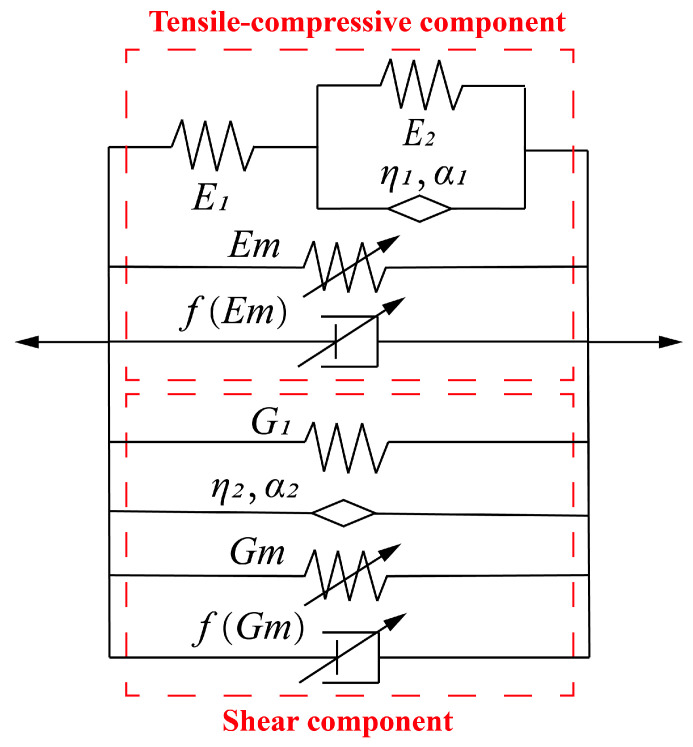
Compression–shear hybrid fractional-derivative parametric model for hybrid-mode MRE isolator.

As shown in Figure 13, the CSHF model appropriately captures the relationship between the mechanical properties of the compression–shear hybrid-mode MRE isolator and both the loading frequency and the applied magnetic field by integrating the tensile–compressive and shear components. The tensile–compressive component consists of a fractional-order derivative Zener (FDZ) model, a variable-stiffness spring element, and a variable-damping viscous element arranged in parallel. The FDZ model is widely employed to simulate the viscoelastic behavior of generalized viscoelastic materials in both the time and frequency domains and can effectively describe the tensile–compressive viscoelastic properties of the MRE matrix. The variable-stiffness spring element and variable-damping viscous element could characterize the magneto-induced tensile–compressive behavior of the MREs. In the tensile–compressive component, $\eta_{1}$ represents the viscosity coefficient, $f\left(E_{m}\right)$ could be assumed to be $\lambda_{c}E_{m}$, and $\alpha_{1}$ denotes an arbitrary fractional-derivative order between 0 and 1. The stress–strain constitutive relation of the tensile–compressive component can be expressed as follows:

(4) $$\frac{E_{1}+E_{2}}{\eta_{1\;}}\sigma_{1}\left(t\right)+D^{\alpha_{1}}\left[\sigma_{1}\left(t\right)\right]+\sigma_{m}\left(t\right)=\frac{E_{1}E_{2}}{\eta_{1\;}}\cdot \varepsilon \left(t\right)+E_{1}D^{\alpha_{1}}\left[\varepsilon \left(t\right)\right]+E_{m}\varepsilon \left(t\right)+\lambda_{c}E_{m}\dot{\varepsilon }\left(t\right)$$

where $E_{1}$, $E_{2}$, and $\lambda_{c}$ are parameters determined by the properties of the MREs, and $D^{\alpha_{1}}$ is the fractional order differentiator. $\sigma \left(t\right)$ and $\varepsilon \left(t\right)$ are tensile–compressive stress and strain varying with time t, respectively. Taking the Fourier transform to Equation (4) yields the following:

(5) $$E^{*}=\frac{\sigma \left(\omega \right)}{\varepsilon \left(\omega \right)}=E_{s}(\omega )+iE_{l}(\omega )$$

where ω is the excitation frequency and $E_{s}$ and $E_{l}$ are the real and imaginary parts of $E^{*}$. Then, the storage and loss modulus of the tensile–compressive component could be obtained as Equations (6) and (7) [46].

(6) $$E_{s}=\frac{E_{1}E_{2}\left(E_{1}+E_{2}\right)+E_{1}(E_{1}+{2E}_{2})\eta_{1\;}\omega^{\alpha_{1}}\cos\left(\frac{\alpha_{1}\pi }{2}\right)+E_{1}{\eta_{1\;}}^{2}\omega^{{2\alpha }_{1}}}{{\left(E_{1}+E_{2}\right)}^{2}+2\left(E_{1}+E_{2}\right)\eta_{1\;}\omega^{\alpha_{1}}\cos\left(\frac{\alpha_{1}\pi }{2}\right)+{\eta_{1\;}}^{2}\omega^{{2\alpha }_{1}}}+E_{m}$$

(7) $$\;E_{l}=\frac{{E_{1}}^{2}\eta_{1\;}\omega^{\alpha_{1}}\sin\left(\frac{\alpha_{1}\pi }{2}\right)}{{\left(E_{1}+E_{2}\right)}^{2}+2\left(E_{1}+E_{2}\right)\eta_{1\;}\omega^{\alpha_{1}}\cos\left(\frac{\alpha_{1}\pi }{2}\right)+{\eta_{1\;}}^{2}\omega^{{2\alpha }_{1}}}+\lambda_{c}E_{m}\omega$$

**Figure 14:**
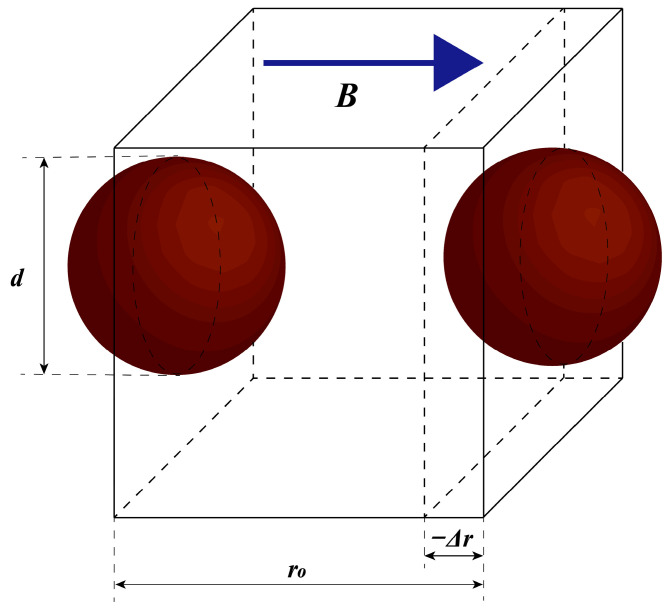
Schematic of dipolar model in tensile–compressive mode.

As for the magneto-induced tensile–compressive modulus $E_{m}$, it could be obtained based on the approach proposed by Jolly et al. [47]. The CIPs are believed to be uniformly distributed in the MRE matrix. When a magnetic field is applied, CIPs polarize in magnetic dipoles and tend to attract each other. The interaction energy W between two dipoles could be expressed as follows:

(8) $$W=\frac{\left|m^{2}\right|(1-3\cos^{2}\left(\theta \right))}{4\pi \mu_{0}\mu_{1}{\left|r\right|}^{2}}$$

where $\mu_{0}$ is the vacuum permeability, $\mu_{1}$ is the relative permeability of the medium ($\mu_{1}=1$), and r is the distance between the two dipoles. θ could be seen as zero under the tensile–compressive mode. As shown in Figure 14, the distance r between the dipoles can be obtained as a function of the axial tensile–compressive strain ε as follows:

(9) $$\varepsilon =\frac{\Delta r}{r_{0}}$$

(10) $$r=r_{0}+\Delta r=r_{0}\left(1+\varepsilon \right)=dh(1+\varepsilon )$$

Therefore, substituting Equation (10) into Equation (8) yields the following:

(11) $$W=\frac{-\left|m^{2}\right|}{2\pi \mu_{0}\mu_{1}d^{3}h^{3}{\left(1+\varepsilon \right)}^{3}}$$

The average interaction energy density of MREs U can be formulated as follows:

(12) $$U=\frac{W\varphi }{V_{i}}=\frac{W\varphi }{(\pi d^{3})/6}=\frac{-3\left|m^{2}\right|\varphi }{\pi^{2}\mu_{0}\mu_{1}d^{6}h^{3}{\left(1+\varepsilon \right)}^{3}}$$

where φ  is the volume fraction of CIPs. Hence, the magnetic-induced tensile–compressive stress can be derived from Equation (12) as follows:

(13) $$\sigma_{magnetic}=\frac{\partial U}{\partial \varepsilon }=\frac{9\left|m^{2}\right|\varphi }{\pi^{2}\mu_{0}\mu_{1}d^{6}h^{3}{\left(1+\varepsilon \right)}^{4}}=\frac{{J_{p}}^{2}\varphi }{4\mu_{0}\mu_{1}{h^{3}(1+\varepsilon )}^{4}}$$

where $J_{p}$ is the dipole moment magnitude per unit particle volume and $\left|m\right|=J_{p}V_{i}=J_{p}(1/6)\pi d^{3}$. Then, the magneto-induced tensile–compressive modulus $E_{m}$ could be formulated as follows:

(14) $$E_{m}=\frac{{J_{p}}^{2}\varphi }{4\mu_{0}\mu_{1}{h^{3}(1+\varepsilon )}^{4}\varepsilon }$$

Then, the tensile–compressive equivalent stiffness and equivalent damping can be expressed as follows:

(15) $$K_{ec}=\frac{E_{s}n_{c}A_{c}}{h_{c}}$$

(16) $$C_{ec}=\frac{E_{l}n_{c}A_{c}}{h_{c}\omega }$$

where $h_{c}$ denotes the height of the compression-type MRE layers, $n_{c}$ represents the number of compression-type MRE layers, and $A_{c}$ means the compressive area of the compression-type MRE layers. The tensile–compressive force $F_{c}(t)$ and the displacement $u_{c}(t)$ could be given by the following:

(17) $$F_{c}\left(t\right)=A_{c}\sigma \left(t\right)$$

(18) $$u_{c}\left(t\right)=h_{c}\varepsilon \left(t\right)$$

The force–displacement relationship of the tensile–compressive component could be expressed as follows:

(19) $$\begin{array}{c} \frac{E_{1}+E_{2}}{\eta_{1\;}}F_{1}\left(t\right)+D^{\alpha_{1}}\left[F_{1}\left(t\right)\right]+F_{m}\left(t\right)=\frac{{A_{c}E}_{1}E_{2}}{{h_{c}\eta }_{1\;}}\cdot u_{c}\left(t\right)+\frac{{A_{c}E}_{1}}{h_{c}}D^{\alpha_{1}}\left[u_{c}\left(t\right)\right]+\frac{{A_{c}E}_{m}}{h_{c}}u_{c}\left(t\right)\\ +\frac{{A_{c}\lambda_{c}E}_{m}}{h_{c}}\dot{u_{c}}\left(t\right) \end{array}$$

This article uses harmonic displacement excitation, $u_{c}\left(t\right)$, which can be written as follows:

(20) $$u_{c}\left(t\right)=u_{0}\sin\left(\omega t\right)$$

The tensile–compressive force $F_{c}(t)$ could he expressed as follows:

(21) $$F_{c}\left(t\right)=\frac{A_{c}E_{s}}{h_{c}}u_{c}\left(t\right)+\;\frac{A_{c}E_{l}}{\omega h_{c}}\dot{u_{c}}\left(t\right)$$

The shear component is composed of a spring element, a nonlinear spring element, a fractional-derivative dashpot element, and a nonlinear viscous element arranged in parallel. The nonlinear spring element and nonlinear viscous element denote the magneto-induced shear modulus of MREs, and the spring element and fractional-derivative dashpot element represent the shear viscoelastic properties of MREs. In the shear component, $G_{m}\;$ is the magneto-induced shear modulus, $f\left(G_{m}\right)$ is expressed by $\lambda_{s}G_{m}$, $\eta_{2}$ represents the viscosity coefficient, and $\alpha_{2}$ denotes an arbitrary fractional-derivative order. Assuming the input strain is $\varepsilon \left(t\right)$ and the output stress is τ(t), the stress–strain relationship could be described as follows:

(22) $$\tau \left(t\right)=G_{1}\cdot \varepsilon \left(t\right)+\eta_{2\;}D^{\alpha_{2}}\left[\varepsilon \left(t\right)\right]+G_{m}\cdot \varepsilon \left(t\right)+\lambda_{s}G_{m}\dot{\varepsilon }\left(t\right)$$

The shear complex modulus $G^{*}$ could be obtained as follows:

(23) $$G^{*}=\frac{\tau (\omega )}{\varepsilon \left(\omega \right)}=G_{s}\left(\omega \right)+i\cdot G_{l}\left(\omega \right)=G_{1}+\eta_{2\;}{\left(i\omega \right)}^{\alpha_{2}}+G_{m}+{i\lambda }_{s}G_{m}\omega \;$$

where $G_{s}$ and $G_{l}$ are the real and imaginary parts of the shear complex modulus. Then, when $i^{\alpha_{2}}=\cos\left(\frac{\alpha_{2}\pi }{2}\right)+i$ $\sin\left(\frac{\alpha_{2}\pi }{2}\right)$ is substituted into Equation (23), the storage and loss modulus of the shear component could be obtained as follows:

(24) $$G_{s}=\;G_{1}+G_{m}+\eta_{2\;}\cos\left(\frac{\alpha_{2}\pi }{2}\right)\omega^{\alpha_{2}}$$

(25) $$G_{l}=\eta_{2\;}\sin\left(\frac{\alpha_{2}\pi }{2}\right)\omega^{\alpha_{2}}+\lambda_{s}G_{m}\omega$$

As for the magneto-induced modulus $G_{m}$, also according to the dipole model proposed by Jolly et al. [47], the magneto-induced shear modulus could be described as Equation (26).

(26) $$G_{m}=\frac{\varphi \cdot (4-\gamma^{2})\cdot J_{p}^{2}}{8\mu_{1}\mu_{0}\cdot {\left(\gamma^{2}+1\right)}^{7/2}}$$

where φ is the volume fraction of CIPs, γ is the shear strain of MREs, and the other parameters are the same as the tensile–compressive MREs. The shear equivalent stiffness and equivalent damping can be expressed as follows:

(27) $$K_{es}=\frac{G_{s}n_{s}A_{s}}{h_{s}}$$

(28) $$C_{es}=\frac{G_{l}n_{s}A_{s}}{h_{s}\omega }$$

where $h_{s}$ denotes the thickness of the shear-type MRE layers, $n_{s}$ represents the number of shear-type MRE layers, and $A_{s}$ means the shear area of the shear-type MRE layers. Then, the final equivalent stiffness ($K_{e}$), energy dissipation ($E_{d}$), and equivalent damping ($C_{e}$) of this model can be expressed as follows:

(29) $$K_{e}=K_{ec}+K_{es}$$

(30) $$C_{e}=C_{ec}+C_{es}$$

(31) $$E_{d}=C_{e}\pi \omega {u_{0}}^{2}$$

The cycle energy of the hysteresis loop is also commonly described by the energy dissipated per cycle, which could be abbreviated as EDC. The formula for the EDC could be expressed as follows:

(32) $$EDC=\pi \cdot F_{max}\cdot X=\pi \cdot c\cdot (2\pi \cdot f)\cdot X^{2}$$

where f denotes the excitation frequency, $F_{max}$ is the maximum force of the hysteresis loop, and c is the viscous coefficient, which is different from the damping ratio *ζ*. Therefore, the viscous coefficient c could be given by the following:

(33) $$c=\frac{EDC}{\pi \cdot (2\pi \cdot f)\cdot X^{2}}$$

It can be clearly seen from Equation (33) that the viscous coefficient c decreases with excitation frequency.

The shear force $F_{s}(t)$ and the displacement $u_{s}(t)$ could be given by the following:

(34) $$F_{s}\left(t\right)=A_{s}\tau \left(t\right)$$

(35) $$u_{s}\left(t\right)=h_{s}\varepsilon \left(t\right)=u_{0}\sin\left(\omega t\right)$$

Then, the shear force $F_{s}(t)$ and the total force of the compression–shear hybrid-mode MRE isolator could be expressed as follows:

(36) $$F_{s}\left(t\right)=\frac{A_{s}G_{s}}{h_{s}}u_{s}\left(t\right)+\;\frac{A_{s}G_{l}}{\omega h_{s}}\dot{u_{s}}\left(t\right)$$

(37) $$F\left(t\right)=F_{c}(t)+F_{s}\left(t\right)$$

The formula for the elastic energy equivalent stiffness should be given as follows:

(38) $$E_{elastic}=K_{e}X^{2}=\int_{0}^{X}F_{x}dx+\int_{X}^{0}F_{x}dx$$

where X denotes the displacement amplitude, and $E_{elastic}$ is obtained by the numerical integration of force over displacement from 0 to X and X to 0.

### 4.2. Parameter Identification

The compression–shear hybrid fractional-derivative parametric (CSHF) model, which combines the tensile–compressive and shear components, could effectively capture the effects of frequency and magnetic flux density on the viscoelastic performance of the hybrid-mode MRE isolator. As part of this model, it is crucial to determine the nine parameters, and test data for 25 different operating states are arbitrarily chosen at a displacement amplitude of 0.15 mm. To ensure for a stable parameter identification, we selected an identification amplitude of 0.15 mm, thereby constraining the strain range to a regime of moderate viscoelastic nonlinearity and minimal loop ellipticity—conditions favorable for the fractional-derivative framework with magneto-elastic coupling. The model takes the displacement u(t) as the entered value and calculates the force F(t) utilizing Equation (37), which is called the model-estimated force, FM. Therefore, these nine parameters are identified using a genetic algorithm through a comparison between the model-calculated force, FM, and the experimental force, FE. In addition, the genetic algorithm minimizes a sum of relative-error terms over all samples in the selected loops, and that the algorithmic details (population size, mutation/crossover rates) follow standard MATLAB (2024) defaults, with a warm-start from a coarse grid search.

### 4.3. Comparison of Theoretical and Experimental Results

**Figure 15:**
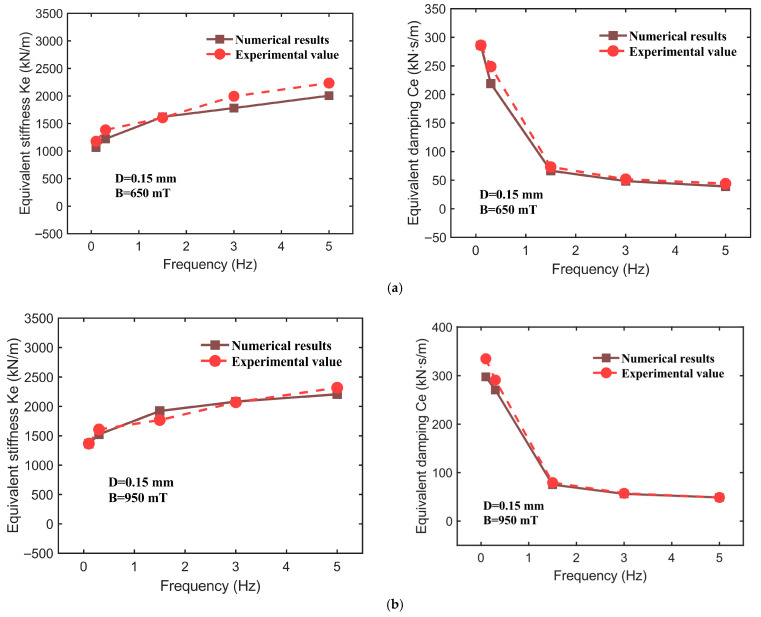
The fitting results for 0.15 mm displacement amplitude at various frequencies. (**a**) Magnetic field strength of 650 mT. (**b**) Magnetic field strength of 950 mT.

**Figure 16:**
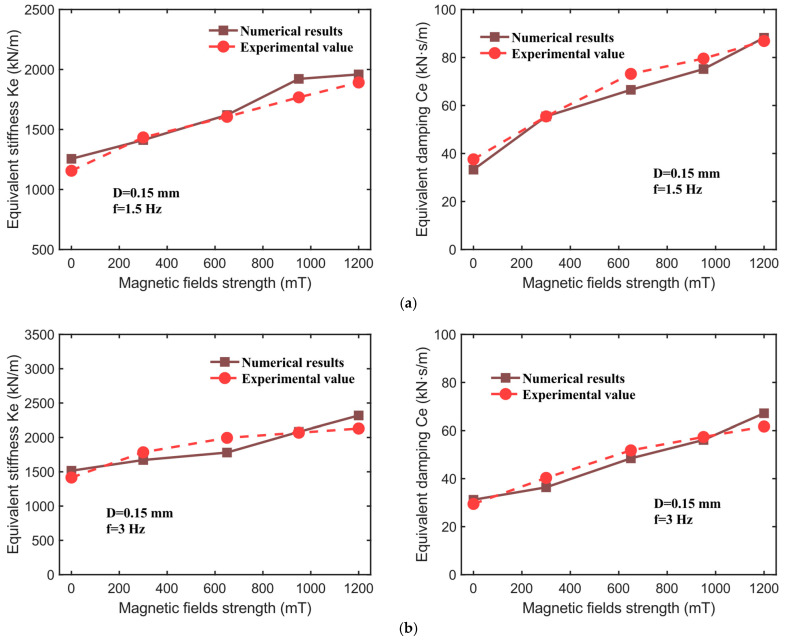
The fitting results for 0.15 mm displacement amplitude at magnetic field strengths. (**a**) Frequency of 1.5 Hz. (**b**) Frequency of 3 Hz.

Based on the experimental data obtained under various operating conditions, this study identifies nine parameters within the CSHF model, with their values listed in Table 2. It is clarified that the identified CSHF parameters represent device-level effective quantities, defined based on the operating state. Parameter B is measured under both compression and shear, and any spatial variation in B between these conditions is implicitly incorporated into the parameters. The dynamic mechanical performance indicators of the hybrid-mode MRE isolator—namely, the effective stiffness $K_{e}$ and effective damping $C_{e}$—are evaluated through a comparative analysis of the model’s computational results and the experimental data, as illustrated in Figure 14, Figure 15 and Figure 16. The corresponding numerical comparisons are provided in Table 3. The comparison in Figure 14, Figure 15 and Figure 16 demonstrates that the CSHF model accurately captures the actual variation trends of each performance indicator, with very small relative error between the model’s predictions and the experimental values.

In Figure 15, for instance, when the displacement amplitude is held constant at 0.15 mm and the magnetic flux density is set to 650 mT and 950 mT, with the excitation frequency varying from 0.1 Hz to 5 Hz, the results are as follows: at a magnetic flux density of 950 mT, the experimental value of effective stiffness $K_{e}$ increases from 1366.18 kN/m (at 0.1 Hz) to 2317.94 kN/m (at 5 Hz), while the model’s predicted values rise from 1364.17 kN/m to 2206.98 kN/m, yielding relative errors of 0.15%, 5.23%, 8.69%, 0.59%, and 4.79%, respectively. Under the same conditions, the relative errors for effective damping $C_{e}$ are 11.07%, 7.04%, 5.51%, 2.19%, and 0.01%. All performance indicators exhibit maximum relative errors below 12%, indicating that the model demonstrates a high precision in describing actual performance characteristics.

Figure 16 illustrates the comparative analysis between the calculated and experimental values of effective stiffness $K_{e}$ and effective damping $C_{e}$ of the hybrid-mode MRE isolator under conditions of a constant displacement amplitude of 0.15 mm, excitation frequencies of 1.5 Hz and 3 Hz, and magnetic flux densities ranging from 0 to 1200 mT. The data in Figure 15 indicate that the CSHF model accurately predicts the dynamic mechanical performance indicators within the 0–1200 mT magnetic field range. For instance, at an excitation frequency of 1.5 Hz, the experimental value of effective stiffness $K_{e}$ increases from 1156.25 kN/m (0 mT) to 1891.63 kN/m (1200 mT), while the model’s calculated values increase from 1255.62 kN/m to 1958.94 kN/m, with relative errors of 8.59%, 1.54%, 0.93%, 8.69%, and 3.56%, respectively. Under identical conditions, the relative errors between the model and experimental values for effective damping $C_{e}$ are 11.47%, 0.01%, 9.09%, 5.51%, and 1.56%. In summary, the CSHF model demonstrates a high degree of accuracy in predicting the dynamic mechanical properties of the hybrid-mode MRE isolator across varying magnetic field intensities and excitation frequencies.

**Figure 17:**
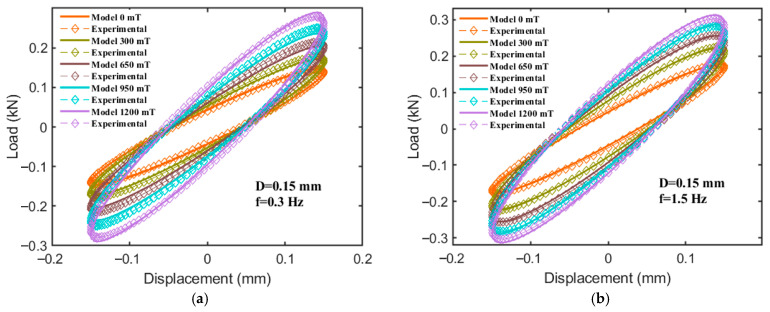
Comparisons between experimental data and model-simulated results. (**a**) f = 0.3 Hz. (**b**) f = 1.5 Hz.

As illustrated in Figure 17, when the displacement amplitude is fixed at 0.15 mm, with excitation frequencies of 0.3 Hz and 1.5 Hz, and a magnetic flux density increasing from 0 to 1200 mT, the force–displacement curves reconstructed by the model for the hybrid-mode MRE isolator exhibit a high degree of conformity with the experimental results. It should be mentioned that the curves in Figure 17 show the model reproducing measured loops using the identified parameters (identification), not an independent validation. The curves generated by both the model and experimental data display an elliptical shape, with the slope and area of the ellipse’s major axis increasing significantly as the magnetic field strength intensifies. This observation further corroborates the superior magnetorheological performance of the hybrid-mode MRE isolator. The comparison of hysteresis curves demonstrates that the CSHF model accurately captures the adjustable stiffness and damping characteristics of the MRE isolator.

## 5. Conclusions

This study presents the design and fabrication of a novel magnetorheological elastomer (MRE) isolator, which operates in a hybrid mode, simultaneously engaging both compression and shear mechanisms. By utilizing a hybrid magnetic field generation system, the magnetic flux density within the MRE isolator is significantly enhanced. To investigate the dynamic mechanical properties of the MRE isolator, an associated dynamic testing system is developed, which facilitates the determination of its equivalent stiffness and damping characteristics. Furthermore, a compression–shear hybrid fractional-derivative parametric (CSHF) model is proposed to describe these properties. A comparison between the experimental results and the model’s predictions reveals key insights, which are summarized in the following conclusions.

(1) Under varying excitation frequencies, when the applied magnetic field strength increases from 0 to 1200 mT, the force–displacement curve of the hybrid-mode MRE isolator exhibits a relatively regular elliptical shape. The slope, fullness, and area of the ellipse’s major axis increase significantly with the magnetic field strength, indicating that the fabricated MRE isolator demonstrates excellent magnetorheological (MR) effects, along with distinctly tunable stiffness and damping characteristics.
(2) The equivalent stiffness ($K_{e}$), energy dissipation ($E_{d}$), and equivalent damping ($C_{e}$) of the hybrid-mode MRE isolator are all influenced by factors such as the excitation frequency, displacement amplitude, and the applied magnetic field. Specifically, the values of all three parameters increase with the applied magnetic field strength. Both $K_{e}$ and $E_{d}$ increase with frequency, while $C_{e}$ decreases as frequency increases. Furthermore, $K_{e}$ and $C_{e}$ significantly decrease with an increasing displacement amplitude, whereas $E_{d}$ increases with displacement amplitude. Regarding the relative MR effect, the MR effects of all three parameters decrease markedly with increasing displacement amplitude and excitation frequency. The maximum MR effect of $K_{e}$ reaches 110.76%, and that of $C_{e}$ reaches 167.98%.
(3) Based on the experimental results, a compression–shear hybrid fractional-derivative parametric (CSHF) model is proposed. The numerical results closely align with the experimental findings, demonstrating the effectiveness of the model in describing the dynamic mechanical properties of the hybrid-mode MRE isolator under varying magnetic field strengths and excitation frequencies.

## Figures and Tables

**Table 1:** Hybrid-mode MRE isolator testing program.

Order	Displacement (mm)	*f* (Hz)	B (mT)
1	0.15	0.1, 0.3, 1.5, 3, 5	0, 300, 650, 950, 1200
2	0.25	0.1, 0.3, 1.5, 3, 5	0, 300, 650, 950, 1200
3	0.4	0.1, 0.3, 1.5, 3, 5	0, 300, 650, 950, 1200
4	0.5	0.1, 0.3, 1.5, 3, 5	0, 300, 650, 950, 1200

**Table 2:** The values of identified parameters.

	Parameters								
	$\alpha_{1}$	$\alpha_{2}$	$E_{1}$	$E_{2}$	$\eta_{1}$	$\eta_{2}$	$G_{1}$	$\lambda_{c}$	$\lambda_{s}$
MRE isolator	0.79	0.77	5.32	0.28	1.99	0.22	2.65	0.036	0.056

**Table 3:** Comparison between numerical and experimental results (D = 0.15 mm).

*H* (mT)	*f* (Hz)	Equivalent Stiffness Ke (kN/m)			Equivalent Damping Ce (kN·s/m)		
		Experimental Value	Numerical Results	Relative Error Value (%)	Experimental Value	Numerical Results	Relative Error Value (%)
650	0.1	1177.272	1063.058	9.702	286.021	286.015	0.002
650	0.3	1385.025	1222.078	11.765	248.714	218.937	11.972
650	1.5	1605.662	1620.655	0.934	73.173	66.523	9.087
650	3	1994.980	1780.590	10.746	51.793	48.458	6.440
650	5	2234.377	2005.878	10.227	44.024	39.110	11.163
0	3	1415.944	1515.554	7.035	29.467	31.199	5.877
300	3	1784.648	1672.012	6.311	40.329	36.385	9.781
950	3	2069.592	2081.695	0.585	57.369	56.112	2.191
1200	3	2129.580	2318.870	8.889	61.690	67.162	8.871
950	0.1	1366.177	1364.164	0.147	334.727	297.670	11.071
950	0.3	1607.267	1523.183	5.231	291.067	270.591	7.035
950	1.5	1767.994	1921.760	8.697	79.565	75.178	5.514

## Data Availability

The raw data supporting the conclusions of this article will be made available by the authors on request.
